# Multi-Epitope Protein as a Tool of Serological Diagnostic Development for HTLV-1 and HTLV-2 Infections

**DOI:** 10.3389/fpubh.2022.884701

**Published:** 2022-05-23

**Authors:** Gabriela de Melo Franco, Anderson Santos da Rocha, Laura Jorge Cox, Danielle Soares de Oliveira Daian e Silva, Débora Marques da Silveira e Santos, Marina Lobato Martins, Luis Claudio Romanelli, Ricardo Ishak, Antonio C. R. Vallinoto, Maria Rosa Q. Bomfim, Adele Caterino-de-Araujo, Jordana G. A. Coelho-dos-Reis, Flávio Guimarães da Fonseca, Edel Figueiredo Barbosa-Stancioli

**Affiliations:** ^1^Laboratório de Virologia Básica e Aplicada, Departamento de Microbiologia, Instituto de Ciências Biológicas, Universidade Federal de Minas Gerais (UFMG), Belo Horizonte, Brazil; ^2^GIPH–Indisciplinary HTLV Research Group, Belo Horizonte, Brazil; ^3^Serviço de Pesquisa, Fundação HEMOMINAS, Belo Horizonte, Brazil; ^4^Laboratório de Virologia, Instituto de Ciências Biológicas, Universidade Federal do Pará (UFPA), Belém, Brazil; ^5^Universidade CEUMA, São Luis, Brazil; ^6^Centro de Imunologia, Instituto Adolfo Lutz, São Paulo, Brazil

**Keywords:** HTLV-1, HTLV-2, serological diagnostic, co-infections, multi-epitope protein

## Abstract

A multi-epitope protein expressed in a prokaryotic system, including epitopes of Env, Gag, and Tax proteins of both HTLV-1 and HTLV-2 was characterized for HTLV-1/2 serological screening. This tool can contribute to support the implementation of public policies to reduce HTLV-1/2 transmission in Brazil, the country with the highest absolute numbers of HTLV-1/2 infected individuals. The chimeric protein was tested in EIA using serum/plasma of HTLV-infected individuals and non-infected ones from four Brazilian states, including the North and Northeast regions (that present high prevalence of HTLV-1/2) and Southeast region (that presents intermediate prevalence rates) depicting different epidemiological context of HTLV-1/2 infection in our country. We enrolled samples from Pará (*n* = 114), Maranhão (*n* = 153), Minas Gerais (*n* = 225) and São Paulo (*n* = 59) states; they are from blood donors' candidates (Pará and Minas Gerais), pregnant women (Maranhão) and HIV+/high risk for sexually transmitted infection (STI; São Paulo). Among the HTLV-1/2 positive sera, there were co-infections with viral (HTLV-1 + HTLV-2, HIV, HCV, and HBV), bacterial (*Treponema pallidum*) and parasitic (*Trypanosoma cruzi, Schistosma mansoni, Strongyloides stercoralis, Entamoeba coli, E. histolytica*, and *Endolimax nana*) pathogens related to HTLV-1/2 co-morbidities that can contribute to inconclusive diagnostic results. Sera positive for HIV were included among the HTLV-1/2 negative samples. Considering both HTLV-1 and HTLV-2-infected samples from all states and different groups (blood donor candidates, pregnant women, and individuals with high risk for STI), mono or co-infected and HTLV-/HIV+, the test specificity ranged from 90.09 to 95.19% and the sensitivity from 82.41 to 92.36% with high accuracy (ROC AUC = 0.9552). This multi-epitope protein showed great potential to be used in serological screening of HTLV-1 and HTLV-2 in different platforms, even taking into account the great regional variation and different profile of HTLV-1 and HTLV-2 mono or co-infected individuals.

## Introduction

The human T-lymphotropic viruses 1 and 2 (HTLV-1 and HTLV-2) are endemic in Brazil that presents the highest number of infected people around the world. According to the recent report of the Brazilian Ministry of Health (1) it was estimated that 800,000 to 2.5 million people are living with HTLV-1/2 in Brazil, with high endemicity variation by state and by distinct groups, as blood donors, pregnant women, and vulnerable populations to sexual transmission infections (0.1–4.8/1,000 inhabitants). The HTLV-2 is either endemic in Brazil among indigenous communities, mainly in the Brazilian Amazon region as well as in injectable drug users in urban areas (2–4). Rates from 1 to 40% were already observed in indigenous populations in isolated communities and epidemiological data showed an HTLV-2 prevalence of 29% in three new villages of the Xikrin tribe (Kayapo indigenous group), showing the greatest prevalence of HTLV-2 around the world (5).

Recently Rosadas and Taylor (6) showed a clear correlation between low income and high HTLV-1 prevalence in pregnant women, which means that virus prevalence increases as economic inequality increases. So, according to the authors, HTLV-1 infection fits the WHO definition of health inequities that are unfair and potentially avoidable with the implementation of adequate policies, with the real possibility to change the landscape of a broad range of high mortality and morbidity diseases caused by HTLV-1. Illustrating this scenario portrayed pregnant women group (due the breast-feeding impact on HTLV-1/2 transmission), 16,548 HTLV-1 women became pregnant by year in Brazil, with the outcome of 3,024 new HTLV-1 infections through the breastfeeding. From those, 120 to 604 and 8 to 272 could develop ATL and HAM/TSP, respectively. Of these infections, 2,610 could be prevented only by counseling for non-breastfeeding in case HTLV was diagnosed during prenatal care (7). Also, in the state of Minas Gerais, Brazil, an HTLV-1/2 screening was conducted using 55,293 dry blood samples from the Neonatal Screening Public-Funded Program, which showed the HTLV-1/2 detection of 9.4/10,000 babies' samples. These cases were confirmed by testing their mothers, indicating a prevalence of 7.6/10,000 represented by 40 HTLV-1 and two HTLV-2 positive mothers. These HTLV-2-infected mothers are from an indigenous Brazilian ethnicity, the Maxacalis tribe which lives in Northeast of Minas Gerais. The North and Northeast regions of Minas Gerais showed the highest prevalence rates (5.59/10,000—Vale do Mucuri and 1.6/10,000—Vale do Jequitinhonha, respectively) and these regions have the state's worst social and economic indicator (8), reinforcing the status of HTLV-1/2 infection as a health inequity. This early finding in mothers contributed to avoid vertical transmission, and therefore this approach using an already well stablished Brazilian Public-Funded Program should be considered as a strategy to avoid HTLV-1/2 transmission. The identification of seropositive individuals is crucial and urgent to block HTLV-1/2 transmission and extend the screening for other groups beyond the blood donors that is already mandatory in Brazil and elsewhere.

One study conducted in Salvador, Bahia, evaluated the performance of four commercially available serological screening tests for HTLV-1 infection in Brazil; three enzyme immunoassays (EIA) and one chemiluminescence assay (CMIA), and concluded that all commercial assays could be safely used (all were 100% sensitive). However, the high sensitivity of some kits may lead to false-positive results, which could increase the testing cost due to the need for confirmation (9, 10), and in the case of blood banks, could increase the discard rate of blood components. In fact, the use of screening assays of high specificity is important to reduce cost with confirmatory assays and prevent distress in people that receive a false-positive result as documented in one study that searched for screening impact in health care programs cost of antenatal screening in Brazil (11). It is important to consider that indeterminate serologic results are the most challenging point in the diagnosis of infectious diseases, impacting different segments of Public Health, and especially the individuals themselves, once it shows to have a direct relation with their health and well-being (12). Thus, due to the high number of WB-indeterminate and untyped HTLV results in HTLV mono or co-infections, it was evaluated the Line immunoassay-LIA in different groups including samples from HIV/AIDS individuals, patients with Hepatitis B and C, both groups from São Paulo and patients from an HTLV outpatient clinic in Salvador, Bahia. LIA confirmed the majority of the WB-indetermined results (66.1, 83.3, and 76%, respectively, in the cited groups). However, the last group from Salvador still presented untyped HTLV results using this technology. Despite the better performance in comparison to WB, the high costs prevented its routine application in Brazil (13).

The International Health Policy Forum for the elimination of HTLV in 2021 (organized by PAHO, WHO, and the HTLV Channel) pointed out the necessity to improve the diagnostic tests, including the production of rapid tests (point of care) to fill the HTLV-1/2 diagnostic gaps and to promote the biotechnology independence in countries with high rates of HTLV-1/2 infection, as in Brazil. In addition, in this period of the COVID-19 pandemic, we are experiencing a shortage of HTLV EIA kits available on the market, joint to the fact that those ready to use were not been tested with samples from different geographic regions and populations in Brazil. Therefore, considering the reasons above and the necessity to provide screening diagnostic tests for HTLV-1/2 in public health services in Brazil, this work aimed at the development of a diagnostic tool based on a multi-epitope recombinant protein containing antigens for HTLV-1 and HTLV-2 that can be used in different diagnostic platforms.

## Methods

### Samples and Ethical Statements

We enrolled samples from the states of Pará, Maranhão, Minas Gerais and São Paulo, Brazil, from blood donors' candidates (Pará and Minas Gerais), pregnant women (Maranhão) and HIV+/high risk for sexually transmitted infection (STI; São Paulo). Among the HTLV-1/2 positive sera, there were coinfections with viral (HIV, HCV, HBV, HTLV-1+ 2), bacterial (*Treponema pallidum*), and parasitic (*Trypanosoma cruzi, Schistosma mansoni, Strongyloides stercoralis, Entamoeba coli, E. histolytica*, and *Endolimax nana*) pathogens related to HTLV-1/2 comorbidities and that might induce inconclusive diagnostic results. The study population consisted of 551 samples of peripheral blood from mono-infected or co-infected individuals with HTLV-1, HTLV-2, or HTLV-untyped, also with other viral and parasitic pathogens, in addition to HTLV seronegative and HTLV seronegative/HIV positive individuals (HTLV-/HIV+) from four different states from Brazil (Table 1). The samples from Minas Gerais (MG) (*n* = 225) were screened at Fundação HEMOMINAS, a blood center in Belo Horizonte, Minas Gerais, by EIA (Murex HTLV-I+II, DiaSorin, Dartford, UK) or microparticle chemiluminescence immunoassay (CMIA Abbott Diagnostics, Germany) and confirmed by Western blot (HTLV BLOT 2.4, MP Diagnostics, Singapore). Serum samples from São Paulo (SP) were provided by Instituto Adolfo Lutz (IAL), a central public health laboratory in São Paulo, and screened by EIA (Gold ELISA HTLV-I+II, REM, São Paulo, SP, Br or Murex HTLV-I+II, DiaSorin, Dartford, UK) and confirmed by WB 2.4. In addition, 114 serum samples from Pará (PA) provided from Universidade Federal do Pará (UFP), and 153 sera from Maranhão (MA) provided from Universidade CEUMA de São Luiz were also tested. Serum samples from Pará were screened by EIA Murex HTLV-I+II, DiaSorin, Dartford, UK, and the positive ones were confirmed by qPCR or nested PCR (*tax* and *env* gene region). Samples from Maranhão were screened by CMIA (Abbott Diagnostics, Germany) and confirmed by WB 2.4 (HTLV BLOT 2.4, MP Diagnostics, Singapore) and nested PCR (*tax* region). The project was approved by the Ethics Committee for Research of Universidade Federal de Minas Gerais (UFMG) and Fundação HEMOMINAS, as well as by IAL, UFP, and CEUMA, receiving the Ministry of Heath protocol numbers CAAE # 55618516.1.0000.5149 and # 55618516.1.3001.5118.

**Table 1:** Characteristics of the HTLV-seropositive and seronegative (SN) sera samples.

	**PA**	**MA**	**SP**	**MG**	**Total**
HTLV-1	28	3	0	66	97
HTLV-1 + HIV	0	0	0	13	13
HTLV-1 + other viral, bacterial and/or parasitic co-infection	0	0	0	15^a^	15
HTLV-2	7	0	0	3	10
HTLV-2 + HIV	0	0	13	3	16
HTLV-2 + other viral and/or parasitic co-infection	0	0	2^b^	2^c^	4
HTLV-untyped and indeterminate	0	2^d^	4^e^	1^f^	7
HTLV- SN / HIV+	49	3	40	0	92
HTLV- SN	30	145	0	122	297
Total	114	153	59	225	551

*States – PA, Pará; MA, Maranhão; SP, São Paulo; MG, Minas Gerais*.

a *HTLV-1 + HTLV-2 (1/15); HTLV-1 + HTLV-2 + HCV + HBV+ Trypanosoma cruzi (1/15); HTLV-1 + HBV (3/15); HTLV-1 + HBV + Treponema pallidum (1/15); HTLV-1 + Treponema pallidum (1/15); HTLV-1 + HCV (1/15); HTLV-1 + HCV + Trypanosoma cruzi (1/15); HTLV-1 + HCV + Strongyloides stercoralis (1/15); HTLV-1 + Strongyloides stercoralis (1/15); HTLV-1 + Trypanosoma cruzi (2/15); HTLV-1 + Schistosoma mansoni (2/15)*.

b *HTLV-1 + HTLV-2 + HIV (2/2)*.

c *HTLV-1/2 HTLV-2 + HBV (1/2); Ascaris lumbricoides, Entamoeba histolytica and Entamoeba coli (1/2)*.

d *WB indeterminate (2/2)*.

e *WB indeterminate (3/4) and untyped (1/4) (all samples are co-infected with HIV)*.

f *HTLV untyped*.

### Multi-Epitope Recombinant Protein Design

A multi-epitope protein was designed for expression in a prokaryotic vector and was based on proteins validated all around the world in EIA and Western blot (WB) tests for HTLV-1 and HTLV-2: Env (gp46) and Gag (p19). In addition, based on our previous studies, Tax protein epitopes were also included for both viruses (14). The chimera's design was based on scientific literature deposited in PubMed [https://www.ncbi.nlm.nih.gov/pubmed/; (14–17)]. Later, the regions of choice were aligned in MultAlin platform (http://multalin.toulouse.inra.fr/multalin/) with Brazilian sequences deposited at GenBank (https://www.ncbi.nlm.nih.gov/genbank/). The chosen epitopes of Env, Gag, and Tax protein were disposed of in tandem, separated by flexible proline and glycine rings (18) and inserted in a commercial pET32a+ plasmid (with histidine tail used for protein purification). *Escherichia coli* Rosetta-gami 2(DE3) cells (Novagen, Merck KGaA, Darmstadt, Germany) were transformed and induced for 5 h with 1 mM IPTG (Sigma-Aldrich, San Luis, Missouri, EUA). The protein was purified with affinity chromatography using sepharose His-Trap HP (GE Healthcare, Chicago, Illinois, EUA) column and Äkta Start system (GE Healthcare Life Sciences, Chicago, Illinois, EUA) and following tested in-house. The multi-epitope protein (1 μg) was transferred to a PVDF membrane (AmershamTM HybondTM 0.45 μM GE Healthcare Life Sciences, Chicago, Illinois, EUA). The membrane was incubated for 16 h at 4°C with a pool of HTLV-1, seronegative, or HTLV-2 infected samples (1:100). A commercial mouse anti-histidine antibody (J099B12–1:1,500–BioLegend, San Diego, Califórnia, EUA) was used as a positive control. As a secondary antibody was used anti-mouse IgG (HAF007–1:10,000–Novus Biologicals, Centennial, Colorado, EUA) or anti-human IgG (A0293–1:2,000–Sigma–Aldrich, San Luis, Missouri, EUA). The detection was performed using a TMB substrate (Sigma–Aldrich, San Luis, Missouri, EUA).

### In-House Indirect ELISA

The sensitivity and specificity of the recombinant multi-epitope protein were tested using an indirect ELISA. The 96-well high-binding flat-bottom microplates (Corning, Nova York, EUA catalog 9018) were coated with the purified recombinant protein (150 ng/well; overnight 4°C) followed by blocking (Low-fat powder milk—LFM—Nestlé, Vevey, Switzerland)-−4 h at room temperature (RT). The microplates were incubated with the sera samples shown in Table 1 (1:50 in wash buffer—LFM 0.1%, tween 20 0.05% in PBS 1 ×; overnight at 4°C) followed by 10 × washing and labeling with secondary antibody anti-IgG human labeled with peroxidase (A0293-−1:25,000—Sigma–Aldrich, San Luis, Missouri, EUA) by 90 min at RT. Human serum (H3667—Sigma–Aldrich, San Luis, Missouri, EUA) was used as negative control and cut-off and TMB (Sigma–Aldrich, San Luis, Missouri, EUA) was used as a chromogenic substrate, followed by reading at 450 nm in Multiskan GO Microplate Spectrophotometer (Thermo Fisher Scientific, Waltham, Massachusetts, EUA). The cut-off was established through the sum of 0.2 factor to the negative control optical density (OD). The index was calculated with the OD of the samples divided by the cut-off. This value was defined using the ROC curve analysis on the preliminary evaluation of the multi-epitope protein parameters, where the OD of the negative control + 0.2 gave the best cut-off related to sensitivity (>95%) observed in the ROC curve. Results above 1.1 were considered positive, below 0.9 negative, and between 0.9 and 1.1 indeterminate.

### Statistical Analysis

Statistical analyses were performed using GraphPad Prism 8.0.2, with Kruskal–Wallis and Mann–Whitney test when appropriate. A receiver operating characteristic (ROC) curve, a statistical tool to evaluate the global accuracy of the test was applied to evaluate the test performance using the same software. Positive and negative predictive values, calculated through the reported prevalence in each state, Youden's *J* statistic, Cohen's Kappa coefficient, and likelihood-ratios were also calculated to evaluate the performance of the multi-epitope recombinant protein to recognize HTLV-1 and HTLV-2 antibodies.

## Results

**Figure 1:**
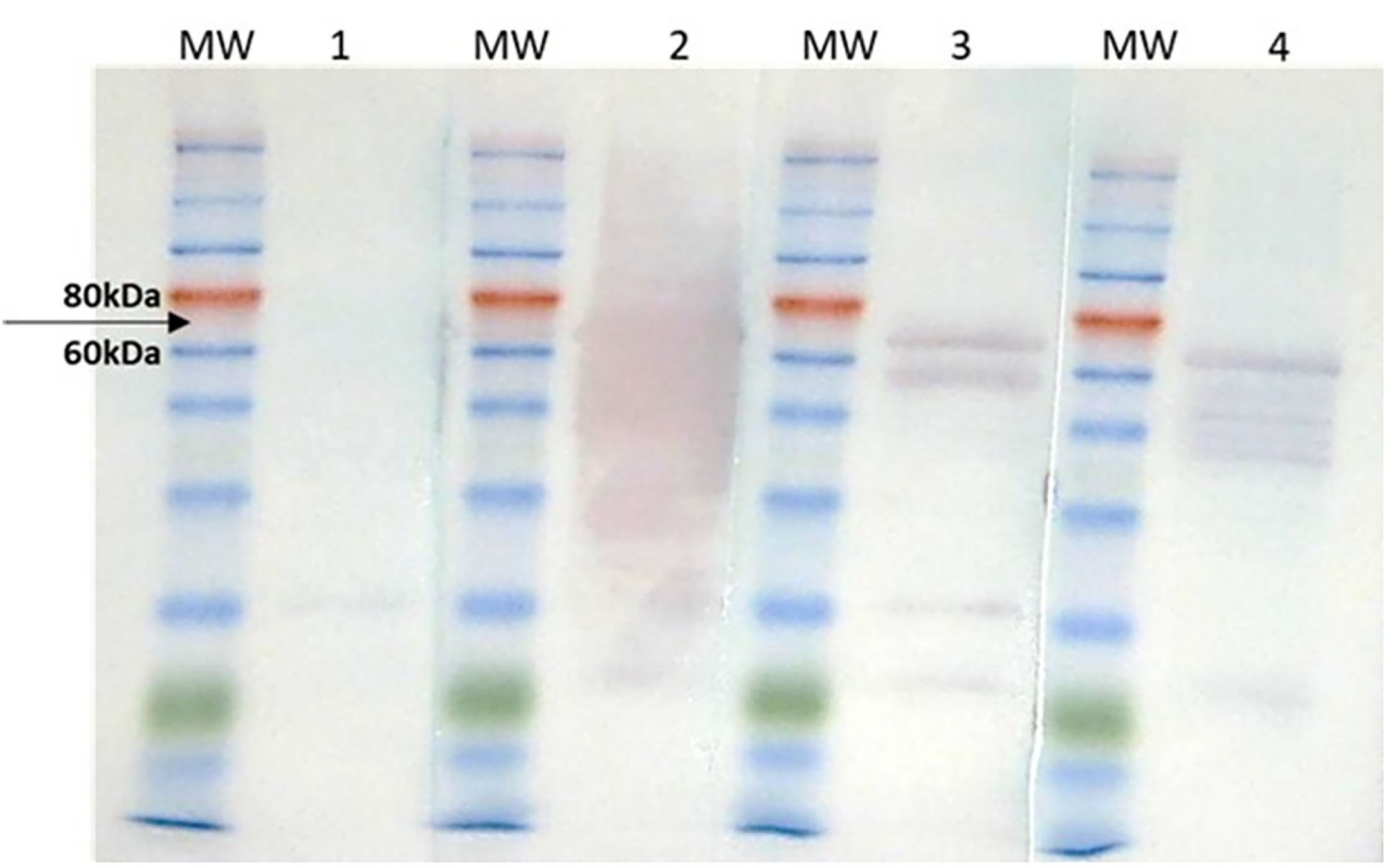
Western blot of purified HTLV-1/HTLV-2 multi-epitope protein. MW, Molecular weight. (1) seronegative pool 1:100/IgG anti-human 1:2,000; (2) positive HTLV-1 pool 1:100/anti-human IgG 1:2,000; (3) positive HTLV-2 pool 1:100 / anti-human IgG 1:2,000; (4) anti-histidine 1:1,500/anti-mouse IgG 1:10,000 (positive control). The black arrow indicates the molecular weight of 70 kDa, corresponding to the size of the recombinant protein. It is observed that the multi-epitope protein was detected by pool sera of individuals infected with HTLV-1 or HTLV-2 (lines 2 and 3, respectively) and by the positive control (line 3—anti-histidine). The pool of seronegative individuals did not react with the target protein (line 1).

The multiepitope protein was designed using HTLV-1 and HTLV-2 p19, gp46, and Tax sequences, and a histidine tail for protein purification by nickel affinity chromatography was added. After the protein purification, a Western blot was conducted. This assay showed that the multi-epitope protein is recognized by sera from individuals infected by HTLV-1 or HTLV-2, which was not observed for the seronegative individuals (Figure 1).

### Characterization of the Multi-Epitope Protein Performance in an In-House Anti-IgG ELISA

**Figure 2:**
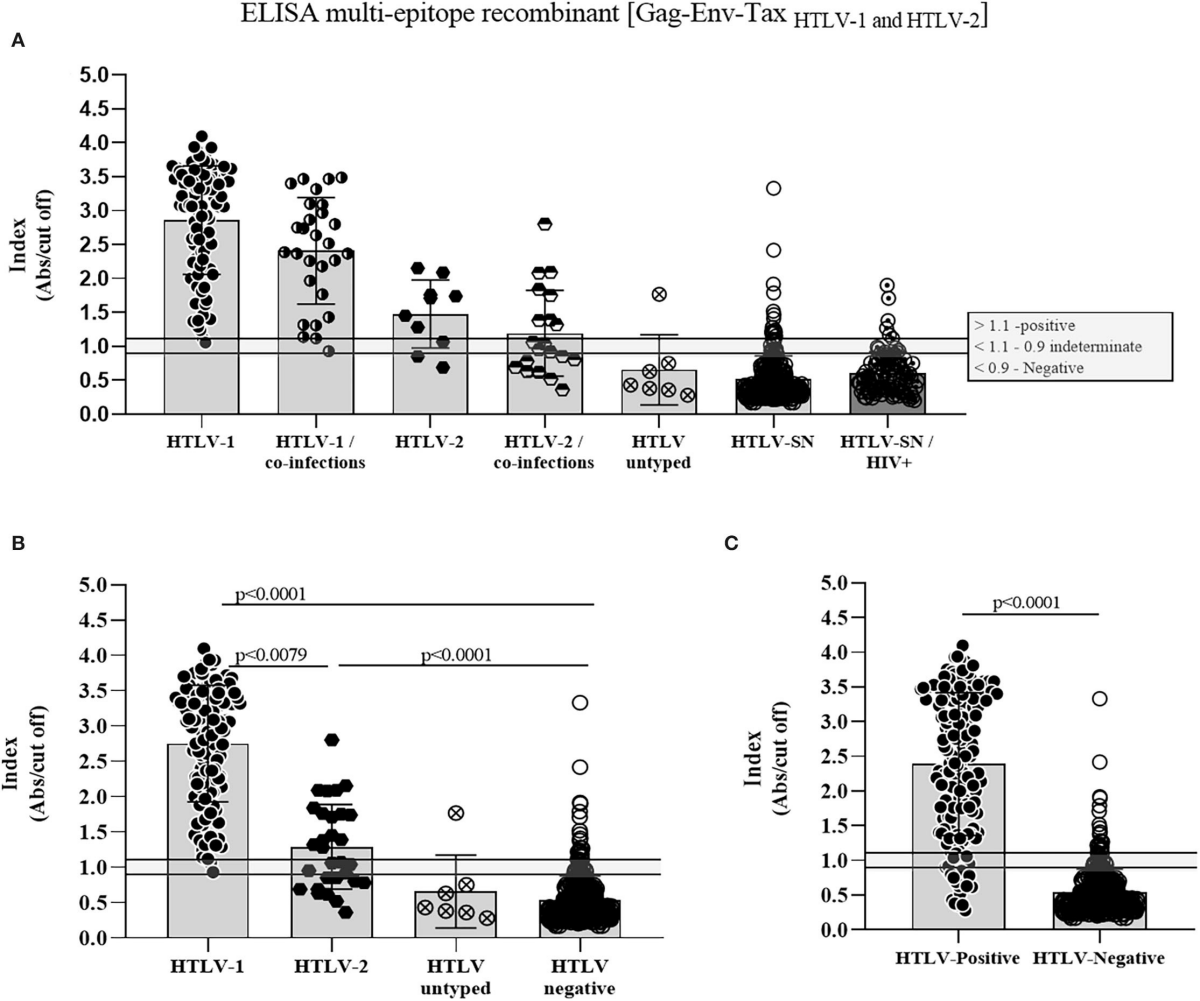
Antibody reactivity of the evaluated HTLV-1, HTLV-2, HTLV, and seronegative samples using the indirect in-house ELISA based on the multi-epitope recombinant protein. **(A)** Scatter plots show the reactivity against the multi-epitope recombinant protein for all samples tested, which were defined as positive, indeterminate, or negative. HTLV-1 mono or co-infected x HTLV-SN or HTLV-SN/HIV (*p* < 0.0001), HTLV-2 mono-infected × HTLV-SN (*p* = 0.0005) and × HTLV-SN/HIV (*p* = 0.00261), HTLV-2 co-infected × HTLV-SN and HTLV-SN/HIV (*p* > 0.05). For HTLV-SN × HTLV-SN/HIV the difference was p = 0.0015. **(B)** Scatter plots comparing HTLV-1/2 groups with HTLV negative. **(C)** Comparison between HTLV positive × HTLV negative. Kruskal–Wallis and Mann–Whitney tests were performed in GraphPad Prism 8.0.2.

The indirect in-house ELISA was performed by double-blind testing of 125 HTLV-1 positive samples (97 mono-infected and 28 with viral coinfection and/or parasitic coinfection), 30 HTLV-2 positive samples (10 mono-infected, 19 with viral co-infection and 1 with parasitic co-infection), two HTLV untyped and five samples with the indeterminate result by serology and 389 HTLV-negative samples, including HIV positive ones (*n* = 92). First, we evaluated the results considering all samples from the four states clustered by viruses: HTLV-1, HTLV-2, and HTLV with or without co-infections (Figure 2A; Table 1). Related to HTLV-1, 2/125 samples (1.6%) showed indeterminate results (1 mono-infected and 1 co-infected sample). On the other hand, from 30 HTLV-2 samples, 6 (20%), and 9 (30%) showed indeterminate and negative results, respectively. Considering the mono-infected and co-infected samples 3.3% and 16.6% were indeterminate, whereas 6.7%, and 23.3% showed negative results, respectively. However, joining HTLV-1 and HTLV-2 samples (mono and co-infected samples), both groups showed statistically significant differences when compared with HTLV negative samples (Figure 2B; *p* < 0.0001 Kruskal–Wallis test). The untyped and indeterminate samples were slightly recognized by the in house-ELISA, with one untyped sample from Minas Gerais State (14.3%) showing a positive results. According to the multi-epitope ELISA, the seronegative controls (*n* = 297) showed 5.1 and 4% of positive and indeterminate results, respectively, whereas these rates were both 6.5% for HTLV-SN/HIV (*n* = 92). Analysis of the negatives samples in a joined group (HTLV-SN and HTLV-SN/HIV+) showed statistically significant differences from HTLV-1 and HTLV-2 (Figure 2B) and from HTLV positive samples (Figure 2C).

### Screening Effectiveness by State's Cohorts

When considering the four different state cohorts examined, it is important to take into account that samples from Pará and Minas Gerais were obtained from blood donors candidates, while the pregnant cohort is originally from Maranhão and the vulnerable population for STI and HIV+ are originally from São Paulo. Results and confidence intervals of all samples analysis are shown in Table 2 (HTLV positive or negative). The sensitivity and specificity results varied among the states. In all states together (named Brazil on the table) the sensitivity was overall 88.27%, with São Paulo (all samples co-infected with HIV) presenting the lowest (47.37%) and Pará the highest value (97.14%). The specificity was 93.06% (Brazil), ranging from 86.08% (Pará) to 97.50% (São Paulo).

**Table 2:** Statistical analysis of the ELISA multi-epitope recombinant's performance by state and HTLV positive and negative samples.

	**Pará**		**Maranhão**		**Minas Gerais**		**São Paulo**		**Brazil**	
	**Value**	**95% CI**	**Value**	**95% CI**	**Value**	**95% CI**	**Value**	**95% CI**	**Value**	**95% CI**
AUC	0.9772	0.9508 to 1.000	0.6926	0.3561 to 1.000	0.9973	0.9939 to 1.000	0.8309	0.7198 to 0.9420	0.9552	0.9345 to 0.9758
Sensitivity	97.14%	85.47% to 99.85%	60%	23.07% to 92.89%	94.17%	87.87% to 97.30%	47.37%	27.33% to 68.29%	88.27%	82.41% to 92.36%
Specificity	86.08%	76.76% to 92.04%	90.54%	84.75% to 94.28%	98.36%	94.22% to 99.71%	97.50%	87.12% to 99.87%	93.06%	90.09% to 95.19%
Cohen's kappa	0.788	0.671 to 0.905	0.234	−0.007 to 0.475	0.928	0.879 to 0.977	0.512	0.277 to 0.747	0.802	0.747 to 0.856
Observed agreements	90.35%		89.54%		96.44%		81.36%		91.65%	
Agreements expected	54.40%		86.35%		50.51%		61.76%		57.89%	
Youden Index	0.83		0.51		0.93		0.45		0.81	
PLR	7.67	4.29 to 13.73	6.34	2.65 to 15.17	57.45	14.52 to 227.29	18.95	2.58 to 138.96	12.72	8.80 to 18.38
NLR	0.03	0.00 to 0.23	0.44	0.15 to 1.29	0.06	0.03 to 0.13	0.54	0.35 to 0.83	0.13	0.08 to 0.19
PPV	6.58%	3.79% to 11.20%	4.28%	1.83% to 9.66%	4.40%	1.15% to 15.40%	22.39%	3.79% to 67.91%	9.28%	6.61% to 12.87%
NPV	99.97%	99.79% to 100.00%	99.69%	99.10% to 99.89%	100.00%	99.99% to 100.00%	99.18%	98.75% to 99.47%	99.90%	99.55% to 99.93%
Prevalence	0.91%		0.70%		0.08%		1.50%		0.80%	

*AUC, Area under the curve; PLR, Positive likelihood ratio; NLR, Negative likelihood ratio; PPV, Positive predictive value; NPV, Negative predictive value; CI, confidence interval*.

*Prevalence: Pará−0.91% - (19); Maranhão−0.7% - (20); Minas Gerais−0.08% - (21); São Paulo−1.5% - (22–24)*.

Cohen's Kappa coefficient measures the reliability of the test, taking into consideration the agreement occurring by chance, in values ranging from 0 to 1, being 1 the perfect agreement (25). Analysis of Minas Gerais' samples resulted in almost perfect agreement (0.928), followed by Brazilian samples overall (0.802) and those from Pará (0.788) with a substantial agreement. Samples from São Paulo showed a moderate agreement (0.512) and from Maranhão a fair agreement (0.234), which is probably related to the fact of that only five HTLV samples from this state were analyzed and two of them (40%) were indeterminate in WB assay.

**Figure 3:**
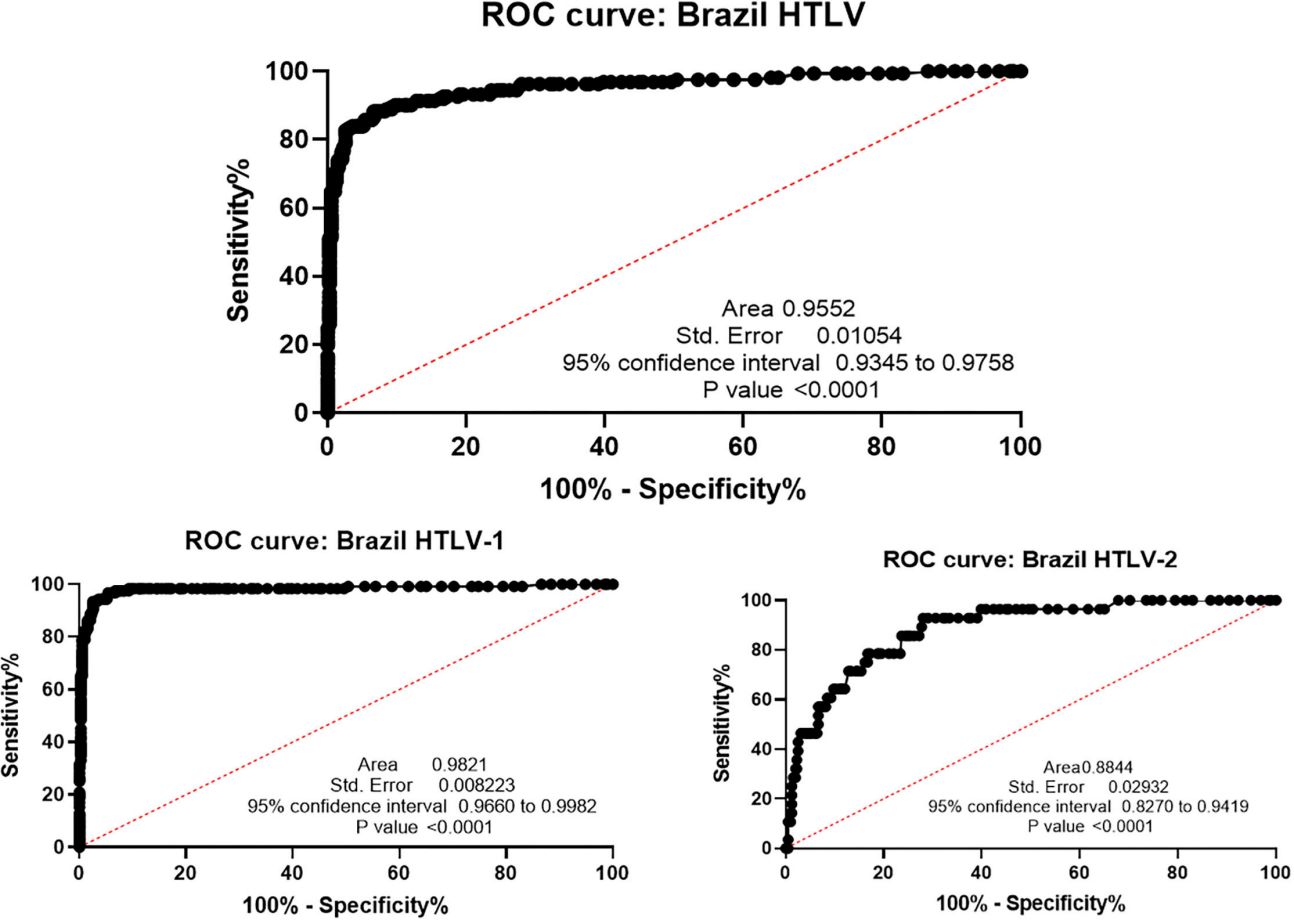
ROC curve analysis of Brazilian global results. HTLV: area under the curve: 0.9552 (95% CI: 0.9345–0.9758), optimal cut-off: >1.110, with 85.80 % sensitivity and 94.60% specificity at cutoff. HTLV-1: area under the curve: 0.9821 (95% CI: 0.9960–0.9982), optimal cut-off >1.110, 96.77% sensitivity, and 94.60% specificity at cutoff. HTLV-2: area under the curve: 0.8844 (95% CI: 0.8270–0.9419), optimal cut-off > 0.9150, 64.29% sensitivity and 90.23% specificity at cut-off.

Both areas under the ROC curve (AUC) and Youden's *J* statistic index indicates accuracy of the test, being 1 the highest accuracy value. The AUC was higher than 0.9 in Minas Gerais (0.9973) and Pará (0.9772), followed by São Paulo (0.8309) and Maranhão (0.6926). Youden's *J* statistic index showed the lowest result in São Paulo (0.51) and the highest in Minas Gerais (0.93). Considering the global result (from the four Brazilian states), the AUC (Figure 3) and the Youden's *J* statistic index were 0.9552 and 0.81, respectively.

Likelihood ratios are used for assessing the value of performing a diagnostic test (26). Values between 0 and 1 indicates the odds of the infection when a test is negative (negative likelihood ratio, NLR) and above 1 indicate the odds of the presence of the infection if the test is positive (positive likelihood ratio, PLR). NLR for São Paulo and Maranhão were 0.54 and 0.44, respectively, while for Pará and Minas Gerais they were 0.03 and 0.06, respectively. The lowest PLR was found in Maranhão (6.34) and the highest in Minas Gerais (57.45). Finally, predictive values indicate the probability of infection if the test is positive (positive predictive value, PPV) and absence of infection if the test is negative (negative predictive value, NPV). As HTLV-1 and HTLV-2 have considered of low prevalence compared to other viral infections, PPV may be underestimated (Pará: 6.58%; Maranhão: 4.28%; Minas Gerais: 4.40%; São Paulo: 22.39%). NPV ranged from 99.18% in São Paulo to 100% in Minas Gerais, being the Brazilian global result 99.90%.

**Figure 4:**
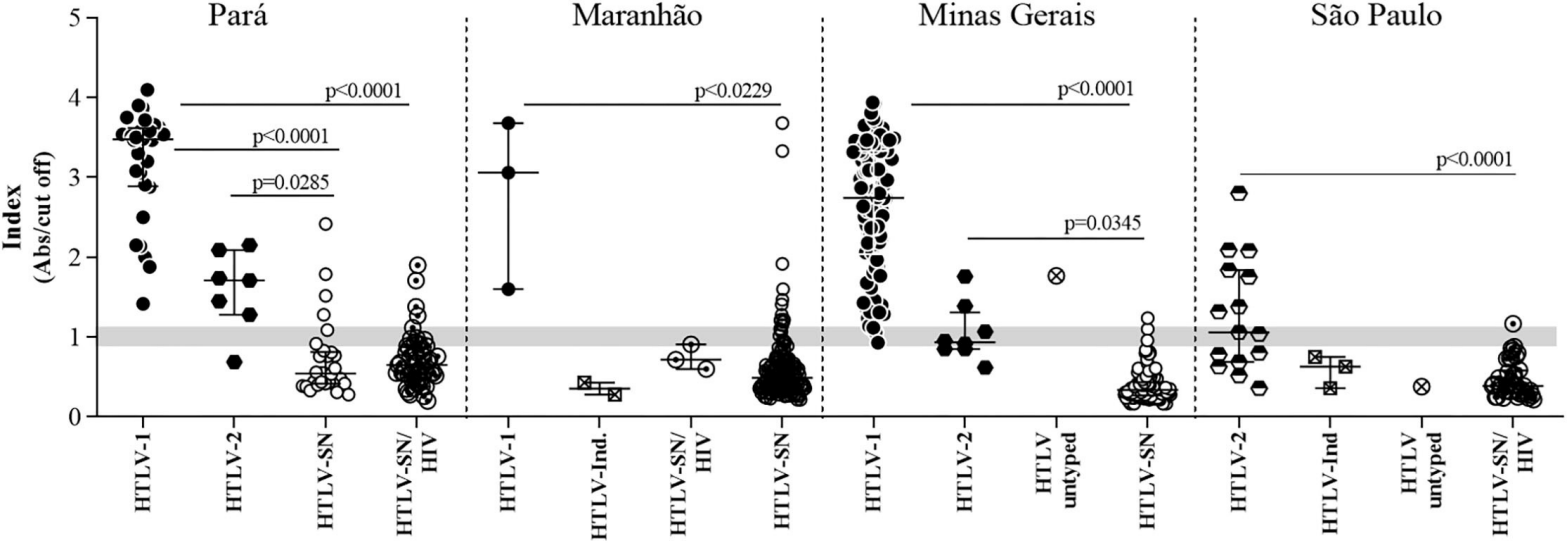
Detection of the multi-epitope chimeric protein by human IgG from individuals infected or not by HTLV in samples from four different Brazilian states. Index values (Abs/cut off) of individuals infected with HTLV-1, HTLV-2, and HTLV and uninfected (SN) in the four states tested—Pará, Maranhão, Minas Gerais, and São Paulo. Ind.: Indeterminate status with CMIA or EIA positive and WB indeterminate pattern (Maranhão and São Paulo). Some HTLV-1 and HTLV-2 samples from Minas Gerais and São Paulo had coinfections, as described in Table 1 and include viral, parasitic and bacterial infections for Minas Gerais and HIV for all samples from São Paulo. HTLV untyped means that WB did not differentiate the infection as HTLV-1 or HTLV-2.

The Index sample's distribution in the ELISA of each state is shown in Figure 4. This screening experimental test differentiates HTLV-positive samples from those negative ones from the four Brazilian states' cohorts (Kruskal–Wallis test), although the group difference of each one (blood donor candidates, low IST risk pregnant women and HIV+/high risk for sexually transmitted infection. The multi-epitope protein was developed with epitopes of HTLV-1 and HTLV-2 and the cut-off evaluated in the data was the same for both viruses (above 1.1 were considered positive, below 0.9 negative, and between 0.9 and 1.1 indeterminate). Figure 3 represents the ROC curve and considering the optimal cut-off > 0.9150 for HTLV-2, the test's performance was 64.29% sensitivity and 90.23% specificity at cut-off for the general Brazilian HTLV-2 samples, showing that including the cut-off should be modified for each virus.

The sensitivity and specificity's results varied among the states, as already shown in Table 2. However, when we analyzed based on samples from each state the regional profile is evidenced (Table 3). Sensitivity regarding to HTLV-1 samples was 100% for Pará and Maranhão and 98.91% for Minas Gerais, with overall value to Brazilian samples 99.19%. The HTLV-1 specificity of detection was overall 92.55%, with Minas Gerais presenting the highest value (98.36%), followed by Maranhão (90.54%) and Pará (86.08%). Maranhão and Pará had the highest number of discordant results related to HTLV seronegative samples (Figure 2) impacting the specificity analyses.

**Table 3:** Statistical analysis of the ELISA multi-epitope recombinant's performance by state and HTLV-1 and HTLV-2 samples.

**HTLV-1**
	**Pará**		**Minas Gerais**		**Maranhão**		**Brazil**	
	**Value**	**95% CI**	**Value**	**95% CI**	**Value**	**95% CI**	**Value**	**95% CI**
AUC	0.9955	0.9882 to 1.000	0.9992	0.9979 to 1.000	0.9932	0.9806 to 1.000	0.992	0.9864 to 0.9976
Sensitivity	100.00%	87.94% to 100.0%	98.91%	94.10% to 99.94%	100.00%	43.85% to 100.0%	99.19%	95.54% to 99.96%
Specificity	86.08%	76.76% to 92.04%	98.36%	94.22% to 99.71%	90.54%	84.75% to 94.28%	92.55%	89.31% to 94.87%
**HTLV-2**
	**Pará**		**Minas Gerais**		**São Paulo**		**Brazil**	
	**Value**	**95% CI**	**Value**	**95% CI**	**Value**	**95% CI**	**Value**	**95% CI**
AUC	0.9042	0.7968 to 1.000	0.9734	0.9441 to 1.000	0.8731	0.7653 to 0.9809	0.8953	0.8404 to 0.9502
Sensitivity	85.71%	48.69% to 99.27%	37.50%	13.68% to 69.43%	53.85%	29.14% to 76.79%	57.14%	39.07% to 73.49%
Specificity	86.06%	76.76% to 92.04%	98.36%	94.22% to 99.71%	97.05%	87.12% to 99.87%	94.61%	90.99% to 96.82%

In regard to HTLV-2, the sensitivity detection was overall 57.14%, with lowest number presented by Minas Gerais (37.50%), followed by São Paulo (53.85%) and Pará (85.71%). The specificity was overall 94.61%; 86.06% in Pará samples, 97.05% in São Paulo, and 98.36% in Minas Gerais.

## Discussion

Serological diagnostic for HTLV-1/2 infections include viral antigens encoded by structural and regulatory genes and methods available fall in two categories: the screening tests and those with the capacity to confirm and discriminate the HTLV-1 and HTLV-2 infections (27, 28). Although efforts have been made around the world to improve the HTLV-1/2 tests, the confirmatory tests are costly and variable in performance according to regional and epidemiological profiles, generating considerable rates of indeterminate and untyped virus results (13, 27, 29, 30).

The diagnostic systems widely used are based on Gag and Env proteins of HTLV-1 and HTLV-2. Gag protein is cleaved into the proteins p15, p19, and p24 that are well characterized as antigens and are present in many commercial assays. Although central and C-terminal portion of Env gp46 showed to be highly immunogenic (31–34), recombinants of Env gp21 present in commercial confirmatory tests are related with false positive and indeterminate results (15–17). A long-term evaluation performed at Fundação Hemominas with 60 blood-donor candidates showed, however, that the reactivity pattern showing only the p24 protein band in Western blot assay occurred in 54.2% of samples, giving indeterminate results (35). Therefore, p24 protein was not considered in the *in-silico* analysis to be incorporated in the multi-epitope protein developed.

The antigenicity of the C-terminal portion of Tax-HTLV-1 was already evidenced using an yeast peptide library (31). Our research group, using an infection-transfection system based on *Vaccinia virus* WR/pLW44 followed by flow cytometric assay showed that the C-terminal portion of Tax protein is highly antigenic and recognize antibodies anti-HTLV-1 in individuals presenting HAM/TSP or rheumatologic diseases and also in asymptomatic carriers (14, 36). Then we developed a multi-epitope protein based on Gag, Env and Tax codifying gene regions for both, HTLV-1 and HTLV-2 viruses to further use in different diagnostic platforms.

The present work is focused in a multi-epitope antigen capable of detecting successfully and simultaneously HTLV-1 and HTLV-2 viruses with sensitivity and specificity and high accuracy, suggesting this antigen as an asset to be used in different serological diagnostic tools. Results showed in here are promising and next steps are in course, including the use of differential peptides based on the same gene regions used in the multi-epitope protein able to distinguish HTLV-1 and HTLV-2.

This multi-epitope protein is expressed in prokaryotic system joining linear epitopes of HTLV-1 and HTLV-2. It was tested in EIA using serum/plasma from four Brazilian states, including the North and Northeast regions (Pará and Maranhão states, that present high prevalence of HTLV-1/2) and Southeast region (Minas Gerais and São Paulo states, that presents intermediate prevalence rates). Nonetheless, to beyond of this regional scope, we enrolled 551 samples from individuals with different profiles, such as blood donors' candidates (Pará and Minas Gerais), pregnant women (Maranhão), and a vulnerable HIV positive individual with a high-risk for sexually transmitted infection (São Paulo). In addition, many of these individuals (32.1% and 23.65% for HTLV seropositive and seronegative, respectively) are co-infected with viral (HIV, HCV, and HBV), bacterial (*Treponema pallidum*) and parasitic (*Trypanosoma cruzi, Schistosma mansoni, Strongyloides stercoralis, Entamoeba coli, E. histolytica*, and *Endolimax nana*) pathogens related to HTLV-1/2 co-morbidities and that can contribute to inconclusive diagnostic results (13, 22, 23, 37–41). Testing samples with these characteristics is important because it covers a diverse population that can be tested in different health services.

Considering both HTLV-1 and HTLV-2 positive samples from all states and different groups (blood donor candidates, pregnant women, and individuals with high risk for STI), mono or co-infected and those HTLV seronegative (including HTLV-/HIV+), the EIA test using the chimeric protein showed a general specificity ranged from 90.09 to 95.19%, and a sensitivity ranged from 82.41 to 92.36% with high accuracy (ROC AUC = 0.9552). The specificity of the assay for HTLV-1 detection was 92.55%, ranging from 89.31 to 94.78%, and the sensitivity was 99.19%, ranging from 99.54 to 99.96 (ROC AUC 0.992). The specificity of the assay for HTLV-2 detection was 94.61%, ranging from 90.99 to 96.82% (ROC AUC 0.8953).

Related to HTLV-1 discordant samples, 1.6% (2/125) positive samples showed indeterminate results in the developed assay. Both are asymptomatic individuals from Minas Gerais (one female and one male). The female is co-infected with *Schistosoma mansoni*. However, this sample was the only one among the 28 co-infected samples that was not positive in the ELISA multi-epitope. On the other hand, there were more discordant results for HTLV-2 positive samples: 6 (20%) and 9 (30%) showed indeterminate and negative results in the ELISA multi-epitope, respectively. Of note, the HTLV seronegative controls (*n* = 297) and HTLV-SN/HIV (*n* = 92) showed 27 and 12 discordant samples, respectively. The Southeast region (Minas Gerais and São Paulo) considered the area of intermediate prevalence in Brazil had 3 and 1 discordant samples, respectively; however, the states Maranhão and Pará that represent the Brazilian North region, the area of highest prevalence in Brazil, presented 20 and 15, respectively. Regarding the discordant results presented, the false-negative samples may be related to their quality (inventory time, storage conditions, and shipment of samples, among others). About the false-positive samples, the developed multi-epitope in-house test was compared with commercial tests used for a long time around the world (and that had already new generation versions) and the necessary test adjustment is in course.

About the false-positive samples, the cross-reactions due to co-infections are already reported by others, and also the developed multi-epitope in-house test was compared with commercial tests used for a long time around the world (and that had already new generation versions) and the necessary test adjustment is course.

Several serological tests have been commercialized, such as ELISA kits from Murex HTLV1+2 (Murex Biotech), Avioq HTLV1/2 (Avioq Inc.), the CMIA Architect rHTLV-1/2 (Abbott Laboratories), and Elecsys® HTLV-1/2 Immunoassay (Roche Diagnostics International Ltd), and as confirmatory assays the WB HTLV Blot 2.4 (MP Biomedicals Asla Pacific Pte Ltd) and the INNO-LIA^TM^ HTLV-1/2 score (Innogenetics® Biotechnology for Healthcare). All of them informing sensitivity and specificity with scores higher than 99% and many reports confirmed around 95% of efficiency in samples from Peru, Sweden, the United States, French, Switzerland, Spain, and Japan (42–44).

In Brazil, da Silva Brito et al. (9) compared the performance of four commercially available serological screening tests for HTLV infection using plasma samples obtained from the biorepository of the Integrated and Multidisciplinary HTLV Center in Salvador, Bahia. Three ELISA kits (Murex HTLV-1/2, Diasorin, anti-HTLV-1/2 SYM Solution, Symbiosis, and Gold ELISA HTLV-1/2, REM), and one CMIA kit (Architect rHTLV-1/2, Abbott) were employed and showed 100% sensitivity, and specificities of >99.5% (SYM and Gold ELISA), 98.1% for Architect, and 92% for Murex. In São Paulo, among HTLV/HIV co-infected individuals, false-positive and false-negative screening results have been described in HTLV-truly infected individuals (23, 41, 45). Although in a low number of samples, negative and/or inconclusive results in screening assays as well a high number of WB inconclusive (indeterminate and HTLV untyped) results were detected; they were solved in part by the confirmatory LIA assay (23, 41). Moreover, the LIA assay confirms HTLV-1 or HTLV-2 positivity in WB-indeterminate samples from individuals with HIV/AIDS (66.1%), HBV and HCV viruses (83.3%) from São Paulo, confirmed HTLV-1 infection also in samples from an HTLV outpatient clinic in Salvador, Bahia (76%), been considered the best serological test to confirming HTLV-1 and HTLV-2 infection (13). Nevertheless, the last group from Salvador still presented HTLV untypable results pointing out that the performance of this commercial test is variable according to cohort specificities tested (12). In HIV/HTLV coinfected individuals, also the molecular tests have low sensitivities in detecting HTLV-1 and HTLV-2 infections, and this finding has been associated with HIV antiretroviral therapy, low HTLV proviral load, point mutations in primers/probes binding regions, and the presence of defective HTLV particles (13, 39, 41, 46).

Recently, a multiplex immunoassay for serological confirmation and differentiation of HTLV-1 and HTLV-2 using samples from French and USA (United State-based cohort HTLV Outcomes Study—HOST) blood banks and also from a Canadian commercial company was reported. This experimental assay is based on GP21, GP46, and p19 having three antigens to detect both viruses and three others to discriminate them. It was developed an algorithm followed by logistic regression formula to characterize each sample and sensitivity and specificity were calculated based on PCR tests and results showed high performance either for diagnostic confirmation or discrimination (22).

Although the current knowledge, the different new approaches developed and in development around the world, and flowcharts for diagnosis based on serological and molecular complementary tests, the HTLV-1/2 diagnostic is still challenging. Our next step, already in course, is test other peptides for the development of a discriminatory rapid tests for HTLV-1 and HTLV-2 using these same gene regions of the multiepitope. The challenge is to obtain a sensitive, specific, low-cost, and easy-to-perform test that can be applied in a variety of health services.

## Data Availability Statement

The original contributions presented in the study are included in the article/supplementary material, further inquiries can be directed to the corresponding author.

## Ethics Statement

The studies involving human participants were reviewed and approved by the Ethics Committees for Research of Universidade Federal de Minas Gerais (UFMG) and Fundação HEMOMINAS, as well by IAL, UFP, and CEUMA, receiving the Ministry of Heath Protocol Numbers CAAE # 55618516.1.0000.5149 and # 55618516.1.3001.5118. The patients/participants provided their written informed consent to participate in this study.

## Author Contributions

EB-S, MM, and GF conceived and designed the experiments. GF, LC, AR, DD, and DS performed the experiments. EB-S, GF, LC, and LR analyzed the data. EB-S, MM, AC-A, MB, RI, AV, JC-R, and FF contributed reagents, materials, and analysis tools. EB-S, GF, MM, and AC-A wrote the paper. All authors contributed to the article, reviewed the manuscript, and approved the submitted version.

## Funding

This work was supported by grant from PPSUS (Programa de Pesquisa para o SUS, Sistema Único de Saúde-Brazilian Unified Health System), Fundação de Amparo à Pesquisa do Estado de Minas Gerais (FAPEMIG-APQ-00907-20), Conselho Nacional de Desenvolvimento Científico e Tecnológico (CNPq), Brazilian Ministry of Health and Pro-Reitoria de Pesquisa da UFMG (PRPq-UFMG). EB-S was supported by PQ#302157/2019-0, RI PQ#312979/2018- 5, and AV PQ#301869/2017-0). FF and GF received fellowship from CNPq. LC and DD (PNPD/CAPES) received fellowship from Coordenação de Aperfeiçoamento de Pessoal de Nível Superior (CAPES).

## Conflict of Interest

The authors declare that the research was conducted in the absence of any commercial or financial relationships that could be construed as a potential conflict of interest.

## Publisher's Note

All claims expressed in this article are solely those of the authors and do not necessarily represent those of their affiliated organizations, or those of the publisher, the editors and the reviewers. Any product that may be evaluated in this article, or claim that may be made by its manufacturer, is not guaranteed or endorsed by the publisher.
